# Uncertainty Quantification for Flow and Transport in Highly Heterogeneous Porous Media Based on Simultaneous Stochastic Model Dimensionality Reduction

**DOI:** 10.1007/s11242-018-1114-2

**Published:** 2018-07-06

**Authors:** D. Crevillén-García, P. K. Leung, A. Rodchanarowan, A. A. Shah

**Affiliations:** 1 School of Engineering, University of Warwick, Coventry, CV4 7AL UK; 2 Department of Materials, University of Oxford, Oxford, OX1 3PH UK; 3 Department of Materials Engineering, Faculty of Engineering, Kasetsart University, 50 Ngamwongwan Rd., Ladyao, Chatuchak, Bangkok, 10900 Thailand

**Keywords:** Porous medium, Dimension reduction, Gaussian process emulation, Spatial fields, Uncertainty quantification

## Abstract

Groundwater flow models are usually subject to uncertainty as a consequence of the random representation of the conductivity field. In this paper, we use a Gaussian process model based on the simultaneous dimension reduction in the conductivity input and flow field output spaces in order quantify the uncertainty in a model describing the flow of an incompressible liquid in a random heterogeneous porous medium. We show how to significantly reduce the dimensionality of the high-dimensional input and output spaces while retaining the qualitative features of the original model, and secondly how to build a surrogate model for solving the reduced-order stochastic model. A Monte Carlo uncertainty analysis on the full-order model is used for validation of the surrogate model.

## Introduction

Groundwater flow models are widely used to study the flow of groundwater and contaminants in soils and aquifers, helping, for example, to mitigate seepage and spillages (Karatzas 2017). Such models, however, are frequently too time-consuming for extensive parametric studies, which has motivated the development of simplified models (Bozic et al. 2009; Vomvoris and Gelhar 1990; Barry et al. 2002).

Of particular interest is the quantification of uncertainties arising from the stochastic representation of the natural heterogeneity of rocks and soils (Nezhad and Javadi 2011; Nezhad et al. 2011; Al-Tabbaa et al. 2000; Kristensen et al. 2010). To date, there have been relatively few attempts at such uncertainty quantification (UQ) (e.g. Feyen et al. 1998; Aly and Peralta 1999; Sreekanth and Datta 2014). Most of the current numerical models used for UQ are based on brute-force Monte Carlo (MC) sampling (Fu and Gomez-Hernandez 2009; Paleologos et al. 2006; Kourakos and Harter 2014; Maxwell et al. 2007; Herckenrath et al. 2011), requiring many runs (of the order 10$^5$) of the numerical model (or *simulator*). For complex simulators, this approach may be impractical unless considerable computing resources are available (Maxwell et al. 2007). Even if such resources are available, they could be better deployed if more efficient methods are developed. This has led to a variety of alternative methods, including analytical models (Gelhar and Axness 1983; Gelhar 1986), multi-grid (or multi-level) algorithms (Giles 2008), surrogate models (emulators) or reduced-order models (Razavi et al. 2012; Ketabchi and Ataie-Ashtiani 2015). The method presented in this paper falls into the latter category.

Data-driven surrogate models have the advantage that no approximation of the physics or numerical scheme is required (they are *non-intrusive*), in contrast to *intrusive* methods that simplify the model and/or reduce the complexity of the numerical scheme, typically *via* projection onto a low-dimensional space. Non-intrusive methods include (generalised) polynomial chaos expansions (Ghanem and Spanos 1991), in which, for instance, the coefficients can be approximated using spectral projection or regression (Xiu and Karniadakis 2002). Such schemes, however, are limited by the input space dimension and polynomial order and tend to perform poorly with limited observations, especially for highly nonlinear problems (Xiu and Hesthaven 2005; Nobile et al. 2008).

Other non-intrusive approaches, also based on data generated from the full model, are based on machine learning methods such as artificial neural networks (ANNs) and Gaussian process (GP) models  (Sacks et al. 1989). Groundwater flow modelling using ANNs is well established (Bhattacharjya and Datta 2005; Kourakos and Mantoglou 2009), but ANNs are not considered to be particularly suited to UQ tasks since they typically require large data sets, as a consequence of fewer *a priori* assumptions. GP models make *a priori* assumptions with regards to the relationship between data points and therefore tend to perform better in cases of limited data, which is an enormous advantage when a simulator is very costly.

GP models have been applied only in a small number of groundwater studies (Bau and Mayer 2006; Hemker et al. 2008; Borgonovo et al. 2012; Crevillen-Garcia 2018). For instance, in Bau and Mayer (2006), the authors use a GP model to learn 4 well extraction rates in a pump-and-treat optimisation problem. In Crevillen-Garcia (2018), the authors measured the gain in computational time of the GP model compared with a highly demanding numerical simulator. In that study, 18 days of continuous intensive CPU computations on a 12-core Intel Xeon cluster processor were required to compute 256 spatial output fields, while only 4h were required to compute the final prediction of the same 256 spatial fields with a GP emulator on the same processor.

In this paper, we are interested specifically in UQ in cases where both the (random) input and output are *fields*, which leads to *high-dimensional* input and output spaces. The original GP modelling framework is impractical for such high-dimensional input and output spaces. To overcome this limitation, we use the empirical simultaneous GP model reduction (ESGPMR) method developed in Crevillen-Garcia (2018). The ESGPMR algorithm is designed to recursively find the lowest dimension of the input space for which the GP emulator response surface best approximates the numerical simulator. The GP emulator is tested on a convection model for which it is possible to perform a full MC UQ to validate the results.

The outline of this paper is as follows. In Sect. 2, we describe the mathematical model, numerical simulator and how we model the uncertainty parameter, namely the hydraulic conductivity. In Sect. 3, we introduce the framework of a GP emulator and the dimension reduction methodology. In Sect. 4, we show and discuss our numerical results and use the MC simulation method for the validation of the approach proposed earlier in Sect. 3. We finish this paper with our concluding remarks.

## Mathematical Model

In this section, we describe the governing equations and the numerical solution of the mathematical model selected for the application.

### Darcy’s Flow in a Horizontal Confined Aquifer

The governing equations used for steady-state, single-phase subsurface flow in a square domain $\mathcal {R} = [0, 1] \times [0, 1]$ consist of Darcy’s law (1) coupled with an incompressible continuity equation (2) (Cliffe et al. 2011, 2000; de Marsily 1986):

1 $$\begin{aligned}&\mathbf {q} + K \nabla h = 0, ~~\mathrm {in}~~ \mathcal {R} \subset \mathbb {R}^2, \end{aligned}$$

2 $$\begin{aligned}&\nabla \cdot \mathbf {q} = 0, ~~\mathrm {in}~~ \mathcal {R} \subset \mathbb {R}^2, \end{aligned}$$

where $$\mathbf {q}$$$$\hbox {m}^2\hbox { s}^{-1}$$ is the Darcy flux, *K*$$\hbox {m s}^{-1}$$ is the hydraulic conductivity, *h* m is the pressure head, and the source terms on the right-hand side of Eq. (2) are set to zero for simplicity. The process considered in this paper is therefore the flow of an incompressible liquid in a horizontal confined aquifer. The governing equations defined in (1) and (2) are coupled to yield a single equation for the pressure head:

3 $$\begin{aligned} \nabla \cdot \left( K(\mathbf{x }) \nabla h(\mathbf{x })\right) = 0,~~~\mathbf {x} = (x, y) \in \mathcal {R}. \end{aligned}$$

The hydraulic conductivity in the above equations characterises the porous medium. Constant values for *K* (homogeneous scenario) would lead to trivial solutions for *h*. In previous studies, it has been shown (see, e.g. Byers and Stephens 1983; Hoeksema and Kitanidis 1985; Russo and Bouton 1992) that spatial variations in the conductivity fields are spatially correlated, and that such fields can be modelled using a log-normal distribution assumption (see, e.g. Laloy et al. 2013; Russo et al. 1994; Russo 1997; Kitterrød and Gottschalk 1997). Thus, in this study we will take the latter approach to model the hydraulic conductivity.

In the next section, we show how to model the hydraulic conductivity as a log-normal random field and how to draw samples. The numerical solution to (3) for a given hydraulic conductivity is then described.

### Generation of Random Conductivity Fields

For any $\mathbf x \in \mathcal {R}$, we can form a real-valued random field indexed by $\mathbf {x}$ on a given probability space $\left( \Omega , \mathcal {F}, \mathbb {P}\right)$, where $Z(\mathbf x , \cdot ): \Omega \rightarrow \mathbb {R}$ is a random variable. For a fixed $\omega \in \Omega$, $Z(\mathbf x , \omega )$ (also written as $Z(\mathbf x )$) is a deterministic function, and when evaluated at all $\mathbf x \in \mathcal {R}$ is called a realisation of the process. We define the mean function $m(\cdot ):\mathcal {R} \rightarrow \mathbb {R}$ of the random field $Z(\mathbf {x})$ by:

$$\begin{aligned} m(\mathbf {x})= \mathbb {E}[Z(\mathbf {x})]=\int _\Omega Z(\mathbf {x})~\mathrm{d}\mathbb {P}(\omega ), \end{aligned}$$

and the covariance function $c(\cdot ,\cdot ){:}\,\mathcal {R}\times \mathcal {R} \rightarrow \mathbb {R}$, by:

4 $$\begin{aligned} c(\mathbf {x}, \mathbf {x}')= \mathbb {E}\left[ (Z(\mathbf {x})-m(\mathbf {x}))(Z(\mathbf {x}')-m(\mathbf {x}')\right] . \end{aligned}$$

In practice, for solving Eq. (3), the numerical model simulator requires the values of the conductivity at the nodes of the discretised domain. Thus, given a set of nodes $\{\mathbf{x }_i\}_{i=1}^M$, the vector $\mathbf {Z}:= (Z(\mathbf {x}_1),\ldots ,Z(\mathbf {x}_M))^{\intercal }$ is a discrete random field. In fact, $\mathbf {Z}:\Omega \rightarrow \mathbb {R}^M$ is a random vector with mean and covariance matrix:

5 $$\begin{aligned} \mathbf {m}=(m_1,\ldots ,m_M)^\mathrm{T}=\mathbb {E}[\mathbf {Z}]\in \mathbb {R}^M, \quad \mathbf C = \mathbb {E} [(\mathbf {Z}-\mathbf {m})(\mathbf {Z}-\mathbf {m})^\mathrm{T}] \in \mathbb {R}^{M \times M}, \end{aligned}$$

respectively, where:

6 $$\begin{aligned} m_i=\mathbb {E}[\mathbf {Z}(\mathbf {x}_i)]=m(\mathbf {x}_i), \quad C_{ij} = c(\mathbf x _i, \mathbf x _j), \quad i,j=1,\ldots ,M \end{aligned}$$

If we now choose $\mathbf {Z}$ to be normally distributed, then $\mathbf {K}= \exp (\mathbf {Z})$ is log normal (Lord et al. 2014).

There are various possibilities for generating Gaussian random fields **Z**, for instance, the circular embedding algorithm (see, e.g. Lord et al. 2014; Dietrich and Newsam 1997; Laloy et al. 2015). While this method provides an exact simulation of a Gaussian random field, the numerical implementation is not trivial and, therefore, it is mainly recommended for extremely large computational domains. A more straightforward method consists of either using a direct Cholesky factorisation or an eigen or Karhunen–Loéve (KL) decomposition of the covariance matrix given in (6) (Strang 2003). These methods also provide an exact representation of the Gaussian field at the grid points, although the computational cost for a large computational domain can sometimes be unaffordable. While a Cholesky factorisation is faster than a eigendecomposition, there are cases in which the method fails due to the strict positive definiteness condition of the numerical scheme (Gill et al. 1996). As a consequence of the characteristics of our mathematical model and the size of the computational domain, in this paper, we opt for the eigendecomposition method (see, e.g. Ghanem and Spanos 1991; Crevillen-Garcia et al. 2017; Crevillen-Garcia and Power 2017). The computational domain does not change over time, and thus the advantage of this approach is that it only requires a single eigendecomposition of the covariance matrix, the results of which are stored and used to generate new realisations of the conductivity field very cheaply.

For modelling the correlation of $\mathbf {Z}$, we use the classical exponential covariance function (see, e.g. Cliffe et al. 2011; Crevillen-Garcia et al. 2017; Hoeksema and Kitanidis 1985; Collier et al. 2014):

7 $$\begin{aligned} c(\mathbf x _i,\mathbf x _j)=\sigma ^{2} ~ \exp \left( \frac{-||\mathbf x _i-\mathbf x _j||_{2}}{\lambda }\right) ~~ \mathbf x _i,\mathbf x _j \in \mathcal {R}, \end{aligned}$$

where $\lambda$ denotes the spatial correlation length and $\sigma ^2$ is the process variance. Appropriate values for these parameters are discussed in Sect. 4. Since the covariance matrix expressed in (6) is real-valued and symmetric, it admits an eigendecomposition (Strang 2003): $\mathbf C = (\Phi \Lambda ^{\frac{1}{2}}) (\Phi \Lambda ^{\frac{1}{2}})^{\intercal }$, where $\Lambda$ is the $M\times M$ diagonal matrix of ordered decreasing eigenvalues $\lambda _1\ge \lambda _2\ge \cdots \ge \lambda _M\ge 0$, and $\Phi$ is the $M\times M$ matrix whose columns $\varvec{\phi }_i$, $i=1,\ldots ,M$, are the eigenvectors of $\mathbf C$. Let $\xi _i\sim \mathcal {N}(0,1)$, $i=1,\ldots ,M$, be independent random variables. We can draw samples from $\mathbf {Z}\sim \mathcal {N}(\mathbf {m}, \mathbf C )$ using the KL decomposition of $\mathbf {Z}$ using the following (Lord et al. 2014):

8 $$\begin{aligned} \mathbf {Z} = \mathbf {m} + \Phi \Lambda ^{\frac{1}{2}}(\xi _1,\ldots ,\xi _M)^{\intercal }= \mathbf {m} + \sum _{i=1}^{M}\sqrt{\lambda _i} \varvec{\phi }_i \xi _i. \end{aligned}$$

The discrete random conductivity field is therefore given by $\mathbf {K} = \exp (\mathbf {Z})$. The terms $\xi _i\sim \mathcal {N}(0,1)$ above will be called *KL coefficients*.

An approximation of $\mathbf {K}$ can be obtained by restricting the expansion in (8) to the first, say, *D* KL coefficients. Although this approximation is commonly used (see, e.g. Cliffe et al. 2011; Kitterrød and Gottschalk 1997), it adds additional uncertainty to the numerical calculations in the form of truncation errors. This also reduces the representation of the heterogeneity yielding to ‘smoother’ conductivity fields. In this paper, we wish to deal with a highly heterogeneous porous medium, and for this purpose, we generate exact realisations of the conductivity field by considering the whole set of *M* KL coefficients (one for each node) when generating conductivity samples. The numerical simulator used to solve Eq. (3) is based on the standard cell-centred finite volume method; then, the only error we have to take into account is the error arising from the numerical (finite volume) scheme. Moreover, the simulator receives as inputs the value of the hydraulic conductivity at the nodes of the computational domain and returns the values of the pressure head at the same nodes. Thus, the simulator can be seen as a mapping from $\mathbf {K}$ to $\mathbf {h}$, where $\mathbf {h} \in \mathbb {R}^{M}$ represents pressure head values at the nodes for a given conductivity input field $\mathbf {K}$. Alternatively, the representation (8) of the Gaussian field allows us to consider a mapping $f_h: \varvec{\xi }\mapsto \mathbf {h}$, for any $\varvec{\xi } = (\xi _1,\ldots , \xi _M)^{\intercal } \in \mathbb {R}^{M}$ distributed according to $\mathcal {N}(\mathbf {0}, \mathbf I )$. In the next section, we will develop an emulator for this mapping.

## Gaussian Process Emulation of Spatial Fields

In this section, we summarise a recent methodology developed in previous work Crevillen-Garcia (2018) for building surrogate models based on GP emulation for a given spatial field simulator, such as the one introduced in Sect. 2. We use GP regression (Rasmussen and Williams 2006), setting a prior specification for the target model by specifying a mean and a covariance function for the GP. The mean and covariance functions are expressed in terms of so-called hyperparameters. This prior distribution is updated by inferring suitable values in the light of data by using the Bayes’ rule. Then, the derived posterior distribution is used for inference. The data used to update the prior distribution are generated by running the numerical simulator at some carefully selected design (input) points and obtaining the simulator outputs (observed values or targets) at these inputs. The data set formed by the design points and the targets is called the training set.

To build the set of design points, we simply spread the points to cover the input space, in this case $\mathbb {R}^M$. There are in the literature several methods for sampling the inputs, for instance, Latin hyper-cube sampling (McKay et al. 1979) or a low-discrepancy sequence (Sobol 1967). We use the latter since it leads to more uniform distributions of points. A more detailed discussion on the different choices of design points can be found in Sacks et al. (1989). The inputs $\varvec{\xi }$ are defined in $\mathbb {R}^M$ and distributed according to $\mathcal {N}(\mathbf{0 }, \mathbf {I})$. Thus, in practice, to form a set of *d* design points, we first generate *d* Sobol points in $[0,1]^M$, and second, we push the *d* points component-wise through the inverse cumulative distribution function of *M* random variables distributed according to $\mathcal {N}(0,1)$, to jointly form the set of design points $\hat{\varvec{\xi }}_j= (\hat{\xi }_j^1,\ldots ,\hat{\xi }_j^M)^{\intercal },~j=1,\ldots ,d$. If we now run the simulator at the design points $\hat{\varvec{\xi }}_j$, we obtain the corresponding observed values $f_\mathrm{h}(\hat{\varvec{\xi }}_j)=\mathbf {h}_j$ to form the training set $\mathcal {D}$.

Finally, for simplicity and without loss of generality, in this study we will use a mean-zero function and the square exponential (SE) covariance function for the prior specification, which is given in terms of hyperparameters as follows (Rasmussen and Williams 2006):

9 $$\begin{aligned} k(\varvec{\xi }, \varvec{\xi }') = \sigma _f^2 \exp \left( -\frac{1}{2}(\varvec{\xi } - \varvec{\xi }')^{\top } \text{ diag }(\ell _1^{-2},\ldots ,\ell _M^{-2})(\varvec{\xi } -\varvec{\xi }') \right) + \sigma ^2_n \delta _{ij}, \end{aligned}$$

where $\sigma _f^2$ is the process variance, $\varvec{\ell } = (\ell _1,\ldots ,\ell _M)$ is the length scale, $\sigma _n^2$ is the noise variance, and $\delta _{ij}$ is the Kronecker delta. The hyperparameters are collectively represented by $\varvec{\theta }= (\sigma _f^2,\varvec{\ell },\sigma _n^2)$. We can make predictions for new untested inputs ${\varvec{\xi }^*}\in \mathbb {R}^M$ by using the predictive equations for GP regression (Rasmussen and Williams 2006):

10 $$\begin{aligned} m_{\mathcal {D}}(\varvec{\xi }^{*})= \Sigma (\varvec{\xi }^{*}, \mathbf X )\left[ \Sigma (\mathbf X , \mathbf X ) + \sigma ^2_n \mathbf I \right] ^{-1}{} \mathbf y , \end{aligned}$$

and

11 $$\begin{aligned} k_{\mathcal {D}}(\varvec{\xi }^{*},\varvec{\xi }^{*}) = k(\varvec{\xi }^{*},\varvec{\xi }^{*})-\Sigma (\varvec{\xi }^{*}, \mathbf X )^{\intercal }\left[ \Sigma (\mathbf X , \mathbf X ) + \sigma ^2_n \mathbf I \right] ^{-1}\Sigma (\varvec{\xi }^{*}, \mathbf X ), \end{aligned}$$

in which $\Sigma (\varvec{\xi }^{*}, \mathbf {X})=(k(\varvec{\xi }^{*}, \hat{\varvec{\xi }}_1),\ldots ,k(\varvec{\xi }^{*}, \hat{\varvec{\xi }}_d))^{\intercal }$. The (*i*, *j*)th entry of $\Sigma (\mathbf {X},\mathbf {X})\in \mathbb {R}^{d \times d}$ is given by $k(\hat{\varvec{\xi }}_i, \hat{\varvec{\xi }}_j)$. Expression (10) for the GP posterior mean $m_{\mathcal {D}}$ can be then used to emulate the simulator output at any new input ${\varvec{\xi }^*}$, i.e. we can write $m_{\mathcal {D}}({\varvec{\xi }^*}) \approx f_{h}({\varvec{\xi }^*})$. Expression (11) provides the predictive variance (error bound) in this estimate of the output.

For high-dimensional input and output spaces, i.e. *M* large, the GP emulation methodology described earlier becomes impractical due to numerical issues when estimating the hyperparameters (Crevillen-Garcia 2018). This necessitates a model reduction technique to reduce the dimension of the input and output spaces to a practical size, while preserving the qualitative features of the original full-order model. In this paper, we will apply to our groundwater flow model the ESGPMR method developed in Crevillen-Garcia (2018) which is described in the next section.

### The Empirical Simultaneous GP Model Reduction Method

The ESGPMR method is designed to overcome the limitation of GPs when dealing with inputs defined in high-dimensional spaces. It also includes a mechanism (the reduced rank approximation) for dimension reduction in the output space. This latter is conducted by using Higdon’s method (Higdon et al. 2008). In this method, the spatial output fields in the training set are projected onto a lower-dimensional space spanned by an orthogonal basis via singular value decomposition (SVD). Thus, the output field can be expressed as a linear combination of principal component analysis (PCA) basis vectors with coefficients treated as independent univariate GPs. In this paper, the accuracy of the reduced rank approximations with respect to the original data will be tested with the $L^2$-norm relative error, i.e. for two vectors $\mathbf{x }= (x_1,\ldots ,x_M)^{\intercal }$ and $\mathbf{y }= (y_1,\ldots ,y_M)^{\intercal }$, we define the $L^2$-norm  relative error between $\mathbf{x }$ and $\mathbf{y }$ as:

12 $$\begin{aligned} \text {RE}(\mathbf{x },\mathbf{y })= \frac{||\mathbf{x }-\mathbf{y }||_{2}}{||\mathbf{x }||_{2}}, \end{aligned}$$

where $||\mathbf{x }||_2$ is the Euclidean norm. The details of the dimension reduction methodology for the output space are given in Crevillen-Garcia (2018), although, for convenience, we reproduce the algorithm below.

Let us consider our simulator $f_h$ which receives inputs in $\mathbb {R}^M$ and returns outputs in $\mathbb {R}^M$ (rather than $\mathbb {R}$). Let $\mathbf {Y}$ be the $M \times d$ matrix with column *j* given by the *j*th run of the simulator.

1. Subtract the mean for each dimension *M* to obtain the centred version $\mathbf {Y}'$ of the matrix $\mathbf {Y}$.
2. Multiply the centred matrix $\mathbf {Y}'$ by the normalisation constant $1/\sqrt{d-1}$ to obtain $\mathbf {Y}^{\prime \prime }$.
3. Compute the SVD of $\mathbf {Y}^{\prime \prime }$ and obtain the $M \times M$ matrix $\mathbf{U}$ whose columns $\mathbf {u}_{j},~j=1,\ldots ,M$, are the PCA basis vectors.
4. Project the original centred data into the low-dimensional space to obtain the matrix of coefficients, $\varvec{\alpha }=(\alpha _{ij}),~i=1,\ldots ,M,~j=1,\ldots ,d$.
5. An orthonormal basis for a lower-dimensional space of dimension $r < M$ is given by the first *r* PCA basis vectors $\{\mathbf {u}_{j}\}_{j=1}^{r}$. Thus, a *reduced rank approximation*$\tilde{\mathbf {Y}}''$ of $\mathbf {Y}''$ can be obtained by using the first *r* columns of $\mathbf{U}$ and the first *r* rows of $\varvec{\alpha }$.

Now, we can build *r* separate and independent GPs from the input space $\mathbb {R}^M$ to $\mathbb {R}$ by generating *r* separate training sets with the coefficients of the PCA basis vector expansion treated as the observed values, i.e. the first *r* rows of $\varvec{\alpha }$. For a new given input ${\varvec{\xi }^*}\in \mathbb {R}^{M}$, we can now employ expression (10) and all of the *r* GPs to estimate the *r* coefficients. These are stored in vector form and can be mapped back to the original output space to obtain the final GP prediction $\mathbf{y }^* \in \mathbb {R}^M$.

Let $\tilde{\mathbf {Y}}^r$ be the reduced rank approximation of $\mathbf {Y}$ obtained by considering the first $r \le M$ coefficients in the PCA basis. The columns $\tilde{\mathbf{y }}_j^r,~j=1,\ldots ,d$, are the corresponding reduced rank approximations of the observed fields $\mathbf{y }_j,~j=1,\ldots ,d$. We wish to reduce the dimension *M* of the original input space. The sequence of training sets is defined as follows: $\{\mathcal {D}_{i}^{D} = (\mathbf {X}^D, \varvec{\alpha }_{i})\}_{i=1}^{r}$, for any $D \le M$, where $\mathbf {X}^D =[ \hat{\varvec{\xi }}_{1}^{D},\ldots , \hat{\varvec{\xi }}_{d}^{D}]$ is the truncated design matrix with *D* of the *M* KL components used (e.g. for $\hat{\varvec{\xi }}_1=(\xi _1^1,\ldots ,\xi _1^D,\ldots ,\xi _1^M)^{\intercal }$ we have $\hat{\varvec{\xi }}_1^D=(\xi _1^1,\ldots ,\xi _1^D)^{\intercal }$), and $\varvec{\alpha }_{i}=(\alpha _{ij}),~j=1,\ldots ,d$. The ESGPMR algorithm (Crevillen-Garcia 2018) is then:

1. Set accuracy tolerance $\varepsilon$ and maximum dimension of the input space to be considered $D_{\max }$.
2. Set $$r=1$$.
3. Find a reduced rank approximation $\tilde{\mathbf {Y}}^r$ of the original $\mathbf {Y}$ by using the first *r* PCA basis vectors.
4. Set $$D=D_{\max }$$.
5. Form the training sets $\{\mathcal {D}_{i}^{D} \}_{i=1}^{r}$ and build *r* independent GPs. Follow the leave-one-out cross-validation (LOO-CV) method and use the GPs to predict the fields at the leave-out points $\hat{\varvec{\xi }}_{j}^{D},~j=1,\ldots ,d$, and check if the following expression holds:
  13 $$\begin{aligned} \text {RE}(\mathbf{y }_j, \hat{\varvec{y}}_{j}^{D}) < \varepsilon ,~ \forall ~j=1,\ldots ,d, \end{aligned}$$
  where $\mathbf{y }_j$ are the columns of $\mathbf {Y}$ (the true fields) and $\hat{\varvec{y}}_{j}^{D}$ denotes the predicted field at $\hat{\varvec{\xi }}_{j}^{D}$.
6. If expression (13) does not hold, set $r=r+1$ and go to (iii) (to refine the reduced rank approximation error). If expression (13) holds, set $D=D_{\max } -1$ and go to (v) (to reduce the dimension of the input space) until the expression does not hold, and then, *returnD* and *r*.

While the value for $\varepsilon$ is set according to the user needs, the value for $D_{\max }$ can be derived from the training data by examining the mean squared error (MSE) as we will see later. To estimate the hyperparameters $\varvec{\theta }= (\sigma _f^2,\varvec{\ell },\sigma _n^2)$ in expression (9), we use the leave-one-out cross-validation (LOO-CV) method (see, e.g. Rasmussen and Williams 2006; Crevillen-Garcia et al. 2017; Crevillen-Garcia 2018). LOO-CV consists of using all the design points of the training set data but one (the *leave-out*) for training, and computing the errors on the predictions for the leave-out points. This process is repeated until all available *d* points have been exhausted. We use each of the *d* leave-out training sets and a conjugate gradient optimiser to obtain estimates of the hyperparameters by maximising the log marginal likelihood (14) w.r.t. the hyperparameters:

14 $$\begin{aligned} \log p(\mathbf y |\mathbf X , \varvec{\theta }) = - \frac{1}{2} \mathbf{y }^{\intercal } (\Sigma + \sigma _{n}^{2}{} \mathbf I )^{-1} \mathbf{y }- \frac{1}{2} \log |\Sigma + \sigma _{n}^{2}{} \mathbf I | - \frac{n}{2} \log 2 \pi . \end{aligned}$$

The prediction errors during the LOO-CV scheme are quantified through the MSE:

15 $$\begin{aligned} \text {MSE} = \frac{1}{d} \sum _{j=1}^{d} (y_j - m_j)^2, \end{aligned}$$

where $m_j$ is the predicted expected value in (10) and $y_j$ the corresponding observed value, both at the same (leave-out) input. In the next section, we apply the dimension reduction and GP emulation techniques introduced earlier to the groundwater flow model described in Sect. 2.1.

## Numerical Results

In subsurface flow applications, $\lambda$ is typically chosen to be significantly smaller than the size of the computational region and also large enough to be taken into account in the numerical formulation (Cliffe et al. 2011). In this paper, we have taken the values from the ranges suggested in the literature (see, e.g. Russo et al. 1994; Russo 1997; Kitterrød and Gottschalk 1997). In order to deal with high heterogeneity we will set a relatively large value for the process variance, $\sigma ^2 = 1.0$. The value for the correlation is set to $\lambda = 0.3$.

Let us consider the mapping $f_h: \varvec{\xi }_j \mapsto \mathbf {h}_j$, for any $j \in \mathbb {Z}^{+}$, which receives as and input the KL coefficients $\varvec{\xi }_j \in \mathbb {R}^{M}$, distributed according to $\mathcal {N}(\mathbf {0}, \mathbf I )$, and used to generate the hydraulic conductivity field $\mathbf {K}_j \in \mathbb {R}^{M}$, and returns as outputs the pressure field $\mathbf {h}_j \in \mathbb {R}^{M}$. To solve Eq. (3) in $[0, 1] \times [0, 1]$, subject to the boundary conditions: $h(0,y)= 100, h(1,y)=0, \frac{\partial h}{\partial y} (x,0)=0, \frac{\partial h}{\partial y} (x,1)=0$, we use a numerical code based on the standard cell-centred finite volume method on a computational grid ($50 \times 50$ centroids) of $M=2601$ nodes (the reader is referred to Cliffe et al. (2011) for full details on the discretisation scheme).

**Fig. 1:**
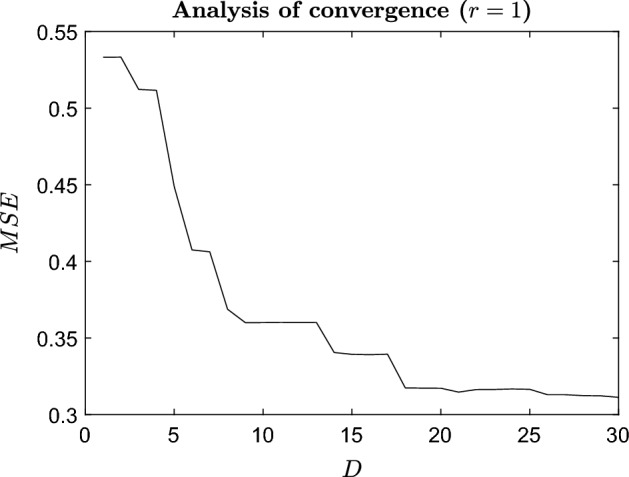
MSE against the number of KL coefficients or input space dimension *D*. This data corresponds to the emulation of the first PC component

**Fig. 2:**
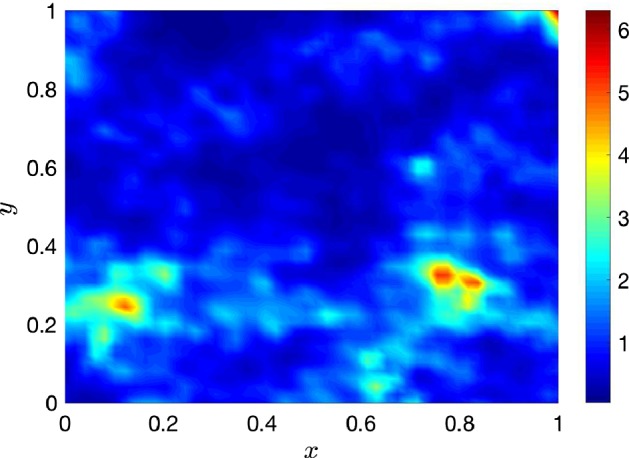
Permeability field used for the prediction of the pressure fields shown in Fig. 3

**Fig. 3:**
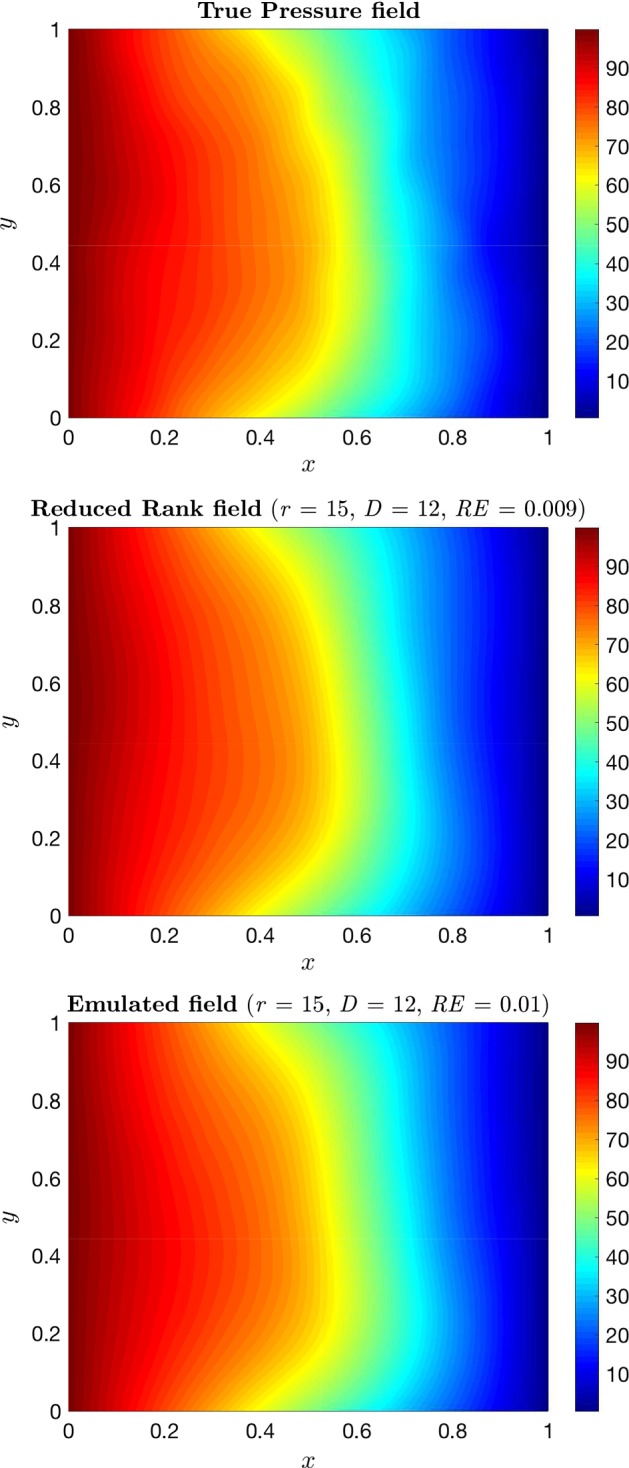
True (top), reduced rank (middle) and predicted (bottom) pressure fields for the conductivity shown in Fig. 2. The dimension of the input (*D*) and output (*r*) spaces and the relative error (RE) achieved are also reported in the pictures

Before we start applying the reduction and emulation techniques, we need to generate some data with the simulator. This will help us to learn the underlying functional form of the model. For doing this, we generate $d=256$ design points $\hat{\varvec{\xi }}_1, \ldots ,\hat{\varvec{\xi }}_d$ from a Sobol sequence as described in Sect. 3. For them, we run our simulator $f_h$ and compute the corresponding pressure fields $\mathbf {h}_j$ to form our training set. Once we have generated the training set, we use the ESGPMR algorithm to reduce the dimensionality of the input and output spaces. Table 1 shows the number of KL coefficients used for the input space, the number of PCs from the PCA basis for the output space and the relative error achieved for different accuracy tolerances $\varepsilon$. From Table 1, we can see that for the larger tolerance $\varepsilon =0.1$, the original problem defined in $\mathbb {R}^{M} \mapsto \mathbb {R}^{M}$ was significantly reduced to $\mathbb {R}^{6} \mapsto \mathbb {R}^{4}$ leading to an overall relative error between true and predicted pressure fields of RE$_{\hbox {true-pred}} = 0.1$. This is already a huge saving in computational cost while keeping a high level of accuracy. And, even for the smallest tolerance considered $\varepsilon =0.01$ the model dimension reduction achieved is $\mathbb {R}^{12} \mapsto \mathbb {R}^{15}$ which is still a substantial reduction from the original dimension $M=2601$ of the input and output spaces. The value of $D_{\max }$ can be estimated by analysing the decay of the MSE for each of the *r* components or by visual inspection. As an example, Fig. 1 shows the decay of the MSE along the input space dimension *D* for $r=1$. In this study, the value of $D_{\max }$ was set to 30. Figures 2 and 3 show, respectively, an example of conductivity for an untested point ${\varvec{\xi }^*}\in {\mathbb {R}^M}$, and the dimension reduction and GP emulation results with $D=12$ and $r=15$ for the same point. The RE between the true and the reduced rank approximation was 0.009. The RE between the true and the predicted was 0.01.

**Table 1:** Relative errors between the true and reduced rank approximation (RE$_{\hbox {true-red}}$) and between and the true and the predicted concentration fields (RE$_{\hbox {true-pred}}$) for three different tolerances ($\varepsilon$)

$\varepsilon$	**PC**	**KL**	$\mathbf{RE }_{\hbox {true-red}}$	$\mathbf{RE }_{\hbox {true-pred}}$
0.100	4	6	0.060	0.100
0.050	8	8	0.035	0.043
0.010	15	12	0.009	0.010

The number of PCs (PC) and KL coefficients (KL) used is also provided

In the next section, we use the reduced-order model obtained for the smallest tolerance ($\varepsilon = 0.01$) investigated earlier, i.e. $D=12$ and $r=15$, to perform a full GP uncertainty analysis on the full-order model. The quantity of interest that will be considered in this application of the ESGPMR method is the travel time of a convected particle in a horizontal confined aquifer.

### UQ of the Travel Time of Convected Particles in Groundwater Flow

**Fig. 4:**
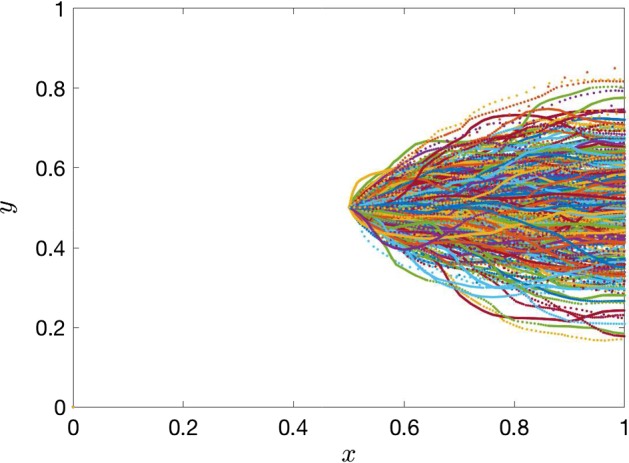
Example of simulated trajectories of a convected particle released at the centre of the domain. These trajectories are used to computed the uncertainty distribution of the travel time $\tau$

The goal is to derive the uncertainty distribution of the travel time $\tau$ that a convected particle (or water molecule) released at the centre of the domain, $(x_0,y_0)=(1/2, 1/2)$, takes to hit the right boundary. To compute the travel time $\tau$, we let $\mathbf {x}=\varvec{\zeta }(t)=(\zeta _{1}(t),\zeta _{2}(t))$ be the location of a particle released from a spatial point $(x_0, y_0)$. After the pressure is calculated for each realisation from Eq. (3), the travel time $\tau$ can be computed by direct Euler integration (Crevillen-Garcia and Power 2017) from the trajectories of the transport equation:

16 $$\begin{aligned} \frac{d\varvec{\zeta }(t)}{dt} = - \frac{K(\varvec{\zeta })}{\phi } \nabla h(\varvec{\zeta }), \end{aligned}$$

subject to the initial condition $\varvec{\zeta }(0)=(x_0, y_0)$, by determining the time $\tau$ for which $\zeta _{1}(\tau )=1$, i.e. when the convected particle lies on the right boundary. A realisation $\mathbf {K}_j$ of the conductivity field represents possible sets of conductivity values in a slice of porous rock across which we would like to study the fluid flow. An example of a set of simulated trajectories for a convected particle for different realisations of the hydraulic conductivity $\mathbf {K}_j$ are shown in Fig. 4. If, for each of the *j* trajectories, we compute the travel time $\tau _j$, we can define the mapping $f_{\uptau }: \varvec{\xi }_j \mapsto \tau _j$, for any $j \in \mathbb {Z}^{+}$, which receives as inputs the KL coefficients $\varvec{\xi }_j \in \mathbb {R}^{M}$ distributed according to $\mathcal {N}(\mathbf {0}, \mathbf I )$ and returns as outputs the travel times $\tau _j \in \mathbb {R}$. To predict the travel times for untested inputs, we can use our GP emulator to predict the pressure fields at the required inputs, and then, derive the predicted travel times as we did with the direct (true) travel times from the transport equation. We can measure the accuracy of the GP emulator predictions by direct comparison with the original simulator $f_{\uptau }$. Next, we perform a MC UQ of the travel time distribution using the numerical simulator. Subsequently, we compare the results to an equivalent UQ using the GP emulator in order to demonstrate its accuracy.

#### Monte Carlo Uncertainty Quantification of the Travel Time Using the Simulator

In this section, we calculate the cumulative distribution function (CDF) of $\tau$, for which we use the MC method (for details on the method, see e.g. Cliffe et al. 2011; Crevillen-Garcia et al. 2017). We use the MC simulation method to approximate the CDF with the empirical cumulative distribution function (ECDF) of a large sample of $\tau$ values as follows: (i) generate a large number *N* of different ensembles $\{\xi _{1,j}^{*},\ldots ,\xi _{M,j}^{*}\}_{j=1}^{N}$ of KL coefficients, where each $\xi _{i,j}^{*}$ is distributed according to $\mathcal {N} (0,1)$; (ii) use the simulator to compute the corresponding true $\tau _j$ for each of the ensembles; (iii) compute the ECDF, $\hat{F}$, of the set of values $\{\tau _j\}_{j=1}^N$ according to:

17 $$\begin{aligned} \hat{F}(s)= \frac{1}{N} \sum _{j=1}^{N} \mathbb {I}_{\{\tau _j \le s\}}, \end{aligned}$$

where $\mathbb {I}$ is the *indicator* function:

$$\begin{aligned} \mathbb {I}_{\{\tau _j \le s\}} = \left\{ \begin{array}{ll} 1~~~~ \mathrm{if}~~ \tau _j \le s,\\ 0~~~~ \mathrm{if}~~ \tau _j > s.\\ \end{array} \right. \end{aligned}$$

**Fig. 5:**
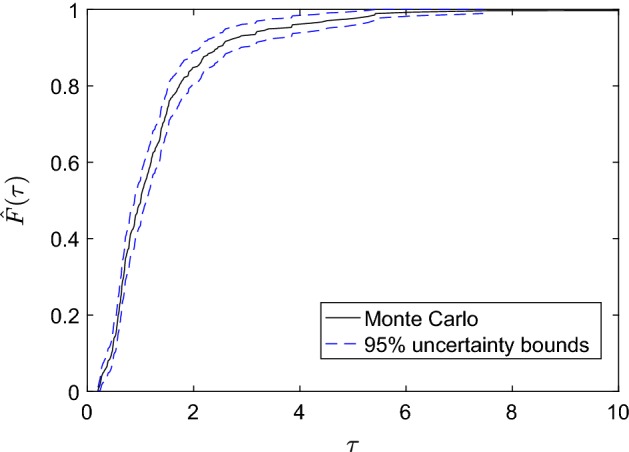
The Monte Carlo ECDF (black line) based on 50,000 travel times from the simulator. The dashed lines show the 95% uncertainty bounds

Figure 5 shows the MC uncertainty analysis for a large sample of $N=50{,}000$ random conductivity fields. The black line is the estimation of the CDF of $\tau$ computed with (17) and the dashed lines the 95% uncertainty bounds for this empirical distribution. The 95% uncertainty bounds are computed by using the Greenwood’s formula implemented in MATLAB^®^ for approximating the variance of the Kaplan–Meier estimator (Cox and Oakes 1984).

#### Gaussian Process Emulation for Uncertainty Quantification of the Travel Time

**Fig. 6:**
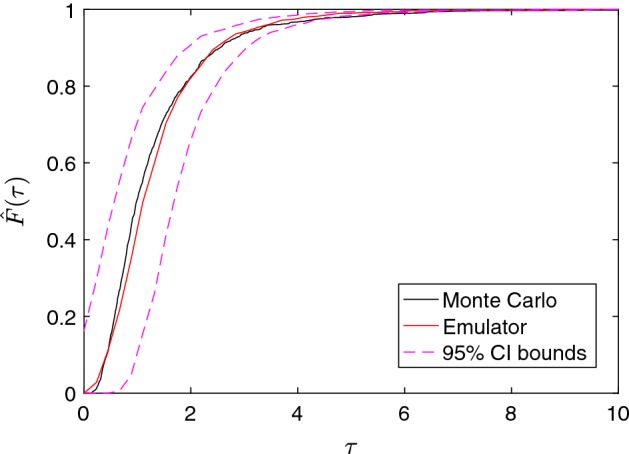
GP uncertainty analysis of the CDF of the travel time based on 50,000 samples

In this section, we use the GP emulator to approximate the distribution of $\tau$ empirically based on the sample size $N=50{,}000$. The idea is to replace the simulator $f_{\uptau }(\cdot )$ by our GP emulator and perform the MC uncertainty analysis as in Sect. 4.1.1. The predictions for untested inputs $\varvec{\xi }_j^*$ and uncertainty bounds are computed by using the predictive mean given by (10). Although a more precise measurement of the accuracy of the GP results could be provided by calculating some analytical scores from the numerical data derived in this study, the goal of this application is to show that the GP emulator is able to quantify the uncertainty at the same level of resolution as MC, and thus, the results of the GP emulation uncertainty analysis are reported in Fig. 6 by direct comparison of both approaches. Figure 6 shows that the ECDF (black) previously computed with the MC method is fully covered with the lower and upper 95% GP uncertainty bounds, i.e. the 2.5th and 97.5th percentiles (dashed magenta). The GP prediction mean (red) of the cumulative distribution function is also provided for reference.

## Conclusions

In this paper, we developed a procedure for quantifying the uncertainty introduced by the randomness of the conductivity (or any other) field on the field output of the groundwater flow model. We used dimension reduction on the input and output fields to develop a feasible routine for Monte Carlo-based UQ. The method was implemented for a model of the travel time of a convected particle in a horizontal confined aquifer, derived from a field output model. The results were compared to a full MC UQ and showed excellent agreement.

Possible extensions of this work to other existing groundwater models include the use on nonlinear dimension reduction techniques, in particular on the output space (Xing et al. 2016, 2015), and the consideration of additional random input parameters (e.g. reaction rates) as an extra source of uncertainty.

## List of symbols

UQ: Uncertainty quantification
MC: Monte Carlo
ANNs: Artificial neural networks
GP: Gaussian process
ESGPMR: Empirical simultaneous GP model reduction
$$\mathcal {R}$$: Physical domain
$$\mathbf {q}$$: Darcy flux
*K*: Hydraulic conductivity
$$\mathbf {h}$$: Pressure head
$$\Omega $$: Sample space
$\mathcal {F}$: Set of events
$\mathbb {P}$: Assignment of probabilities to the events
$\mathbf {Z}$: Gaussian discrete random field
KL: Karhunen–Loéve
$\mathbf {m}$: Expected value of $\mathbf {Z}$
$\mathbf {C}$: Covariance matrix for $\mathbf {Z}$
*c*: Correlation function
$$\sigma ^2$$: Correlation variance
$\lambda$: Spatial correlation length
*M*: Number of grid points
$\Phi$: Matrix of eigenvectors
$\Lambda$: Matrix of eigenvalues
$$\xi $$: KL coefficients
*D*: Dimension of the input space
$f_\mathrm{h}$: Numerical simulator for the pressure head
$$\tau $$: Travel time
$f_{\uptau }$: Numerical simulator for the travel time
*d*: Number of design points
$$\hat{\varvec{\xi }}$$: Design points
SE: Square exponential
*k*: Square exponential covariance function
$$\sigma _\mathrm{f}^2$$: GP variance
$\varvec{\ell }$: GP length scale
$\sigma _\mathrm{f}^2$: GP noise variance
$$\hat{\varvec{\xi }^{*}}$$: Untested inputs
$\varvec{\theta }$: Collective representation of the hyperparameters
$$\mathcal {D}$$: Training set
$$m_{\mathcal {D}}$$: Predictive mean
$$k_{\mathcal {D}}$$: Predictive variance
$$\delta _{ij}$$: Kronecker delta
SVD: Singular value decomposition
PC: Principal component
PCA: Principal component analysis
RE: Relative error
$D_{\max }$: Maximum dimension considered by the ESGPMR method
*r*: Number of PCA basis vectors
MSE: Mean squared error
LOO-CV: Leave-one-out cross-validation
$\varepsilon$: Accuracy tolerance of the ESGPMR method
$\mathbf{RE }_{\hbox {true-red}}$: RE between the true and the reduced rank approximation
$\mathbf{RE }_{\hbox {true-pred}}$: RE between the true and the predicted
*t*: Time
$\varvec{\zeta }$: Location of the convected particle at a given time
*N*: Number of samples
CDF: Cumulative distribution function
ECDF or $\hat{F}$: Empirical cumulative distribution function
$$\mathbb {I}$$: Indicator function
